# Functional proteomic profiling reveals KLK13 and TMPRSS11D as active proteases in the lower female reproductive tract

**DOI:** 10.12688/f1000research.16255.2

**Published:** 2018-12-31

**Authors:** Carla M.J. Muytjens, Yijing Yu, Eleftherios P. Diamandis

**Affiliations:** 1 Department of Laboratory Medicine and Pathobiology, University of Toronto, Toronto, Canada; 2 Department of Pathology and Laboratory Medicine, Mount Sinai Hospital, Toronto, Toronto, Canada; 3 Department of Clinical Biochemistry, University Health Network, Canada, Toronto, Canada

**Keywords:** cervical-vaginal fluid, serine proteases, activtiy-based probe proteomics

## Abstract

**Background**: Cervical-vaginal fluid (CVF) hydrates the mucosa of the lower female reproductive tract and is known to contain numerous proteases. The low pH of CVF (4.5 or below in healthy women of reproductive age) is a uniquely human attribute and poses a challenge for the proteolytic functioning of the proteases identified in this complex biological fluid. Despite the abundance of certain proteases in CVF, the proteolytic activity and function of proteases in CVF is not well characterized.

**Methods**: In the present study, we employed fluorogenic substrate screening to investigate the influence of pH and inhibitory compounds on the proteolytic activity in CVF. Activity-based probe (ABP) proteomics has evolved as a powerful tool to investigate active proteases within complex proteomes and a trypsin-specific ABP was used to identify active proteases in CVF.

**Results**: Serine proteases are among the most abundant proteins in the CVF proteome. Labeling human CVF samples with the trypsin-specific ABP revealed serine proteases transmembrane protein serine 11D and kallikrein-related peptidase 13 as active proteases in CVF. Furthermore, we demonstrated that the proteolytic activity in CVF is highly pH-dependent with an almost absolute inhibition of trypsin-like proteolytic activity at physiological pH levels.

**Conclusions**: These findings provide a framework to understand proteolytic activity in CVF. Furthermore, the present results provide clues for a novel regulatory mechanism in which fluctuations in CVF pH have the potential to control the catalytic activity in the lower female reproductive tract.

## Introduction

Approximately 2–4% of the genes in the human genome account for proteases, and in addition, over 150 different protease inhibitors are encoded in the human genome ^1^. Proteases irreversibly modify proteins via peptide bond hydrolysis and thereby impact on both protein structure and function. As a result, proteases are implicated in nearly every significant biological process.

Cervical-vaginal fluid (CVF) hydrates the mucosa of the vagina and lower part of the cervix. Situated directly at the interface between the external environment and the internal milieu, CVF plays key roles in sexual intercourse, conception and provides a barrier against microbial invasion ^2– 6^. Almost 8% of all proteins identified in CVF are proteases, which highlights the tremendous proteolytic potential of this complex biological fluid ^7^. Interestingly, human CVF differs from other mammals in that it is uniquely acidic with levels of 4.5 or less in healthy women of reproductive age ^8, 9^. The low pH is estrogen dependent and results from the conversion of glycogen to lactic acid by the vaginal microbiome or, alternatively, the vaginal lumen is acidified via active proton secretion across the vaginal epithelial membrane ^10– 12^. The acidity levels in CVF pose a substantial challenge for protease activity in this environment. Despite the high abundance of proteases in CVF, their activity and functioning in the lower female reproductive tract remains to be elucidated.

Functional proteomics using an activity-based probe (ABP) coupled to mass spectrometry has emerged as a powerful tool to study active proteases within complex proteomes ^13, 14^. ABPs, designed to target certain classes of active proteases, have been successfully applied to investigate ABP-mediated profiling of complex proteomes, inhibitor screening and enzymatic target identification ^15, 16^.

In the current study, ABP profiling of CVF resulted in the identification of transmembrane protein serine 11D (TMPRSS11D) and kallikrein-related peptidase 13 (KLK13) as active trypsin-like proteases. In addition, we observed a near complete cessation of trypsin-like proteolytic activity at physiological pH levels in CVF. Therefore, we propose a novel mechanism in which fluctuations in CVF pH have the potential to regulate the catalytic activity in the lower female reproductive tract. Understanding the protease activity and functioning in CVF can provide important insights in their mechanism of action and ultimately lead to novel protease targeted therapeutics.

## Methods

### Sample collection

CVF samples were collected from 10 healthy, non-pregnant female volunteers using a softcup collection device (Instead Inc. San Diego, CA) as described previously ^7, 17^. Informed consent was obtained from all participants prior to sample collection. The collection and use of human samples was approved by the Research Ethic Board at Mount Sinai Hospital in Toronto (MSH REB 16-0137-E). All women abstained from sexual activity and douching at least 3 days prior to sample collection and had no self-reported vaginal infections. The sample was collected after 90 minutes via centrifugation (Eppendorf centrifuge, model 5415R) of the softcup inside a 50mL conical tube at 200xg for 2 minutes. CVF was transferred to a 1.5mL tube and centrifuged at 15,000xg for 20 minutes to remove cellular debris. Total protein of each CVF sample was determined using a Bradford protein assay (Thermo Fisher Scientific, #1856209)). The samples were stored at -80°C until further use.

### Estimation of protease abundance in CVF proteome

The estimation of protease abundance in the CVF proteome was based on the CVF proteome dataset from healthy non-pregnant women ^7^. Proteases were identified by comparison with the Merops database and grouped according to their catalytic type ^1^. The log transformed MS1 area data were used as a proxy for the relative abundance of each protein within the dataset. CVF proteins and proteases were subsequently ranked according to the relative abundance and plots were produced using R (R version 3.5.0).

### Investigation of proteolytic activity in CVF

The trypsin-like proteolytic activity in CVF was measured by incubating 0.1µg total protein CVF sample with 0.5mM of the fluorogenic t-butoxycarbonyl-tri-peptide-7amino-4-methylcoumarin (AMC) synthetic peptide (Bachem, #4003460) with a valine (V), proline (P), arginine (R) tripeptide sequence. Fluorescence was measured every minute for 15 minutes on a Wallac Envision fluorometer (Perkin-Elmer) set at 355nm for excitation and 460nm for emission. All reactions were performed at 37°C in female reproductive tract buffer (20mM potassium phosphate, 60mM sodium chloride) ^18^. To determine the influence of pH on the proteolytic activity in CVF the buffers were adjusted using NaOH and HCl so that pH ranged from 9.0 to 3.5 in 0.5 increments. Trypsin-like proteolytic activity in CVF was also measured in the presence of 1 mM phenylmethylsulfonyl fluoride (PMSF), 10mM ethylenediaminetetraacetic acid (EDTA) or 5nM soybean trypsin inhibitor (STI). Proteolytic activity was quantified by calculating the rate of substrate hydrolysis after subtraction of the fluorescence of enzyme-free reactions. All experiments were performed in triplicate on 2 separate days. Figures were prepared using GraphPad Prism (version 7.00 for Windows).

### Visualization of trypsin-like proteolytic activity in CVF

A trypsin-specific, biotin-conjugated activity-based probe (ABP) was used to label the active enzymes in CVF specifically and covalently ^19^. The probe was kindly provided by Professor Brendon Gilmore (Queen’s University Belfast, Ireland). Active enzymes were labeled by incubating 1 µg total protein of CVF sample in 5 µL (in 10mM Tris, pH 8.0) with 1 µL 10x proteinase assay buffer (500mM Tris-HCl, pH8.0, 2% NP40, 15mM CaCl _2_), 1 µL of 1 mM ABP and 2 µL H _2_O. Additionally, CVF sample was incubated with 10mM EDTA, 1mM PMSF and 5nM STI prior to the labeling of the active enzymes with the ABP. After a 2-hour incubation at room temperature, the reaction was stopped using SDS sample buffer (31.25mM Tris-HCl pH 6.8, 12.5% glycerol, 1% SDS, 0.005% Bromphenol Blue (Biorad, #161-0747) containing 50mM DTT, followed by boiling for 10 minutes to denature the proteins. Samples were loaded on a 4–15% Mini-Protean TGX precast gel (Biorad, #456-1086/1083) and SDS gel electrophoresis was performed at 200V for approximately 30 minutes. Proteins were transferred using a 0.2 µm PVDF membrane (BioRad, #1704156) in a Trans-blot Turbo Blotting System (Biorad). The membranes were blocked with 5% casein in TBS-T (0.05% Tween-20 in 50mM Tris, 150 mM NaCl, pH 7.5) at room temperature for 2 hours, followed by a wash with TBS-T (3 × 10 minutes). The membrane was subsequently incubated with streptavidin-horseradish peroxidase (SA-HRP) (Jackson Immunoresearch, #016-030-084) in a 1:10,000 dilution in 1% casein in TBST-T for an hour, followed by an extensive wash with TBS-T (3 × 15 minutes, 3 × 5 minutes). A total of 125 µL of chemiluminescence substrate (GE healthcare, #45000875) was added per square centimeter of membrane. The membrane was then placed into an autoradiography cassette, exposed for 6 minutes and developed using x-ray film.

### Separation of high and low molecular weight active trypsin-like proteases

CVF samples (N= 5) were fractionated using size exclusion chromatography (SEC) in order to isolate the fractions with demonstrated proteolytic activity. A total of 250µg total protein per sample was loaded onto a TSK gel G3000SW column (Tosoh Bioscience) in 0.1M Na _2_HPO _4_/Na _2_SO _4_ (pH 6.8) and fractionated with a constant flow rate of 0.5mL/min. Fractions were collected every minute throughout the gradient and subsequently analyzed for proteolytic activity using the fluorescent substrate and ABP as described previously. The trypsin-like proteolytic activity in the SEC fractions was quantified via comparison to standard curve prepared with porcine trypsin (Sigma, 16,000 BAEE U/mg, #T0303).

### Identification of active trypsin-like proteases in CVF via mass spectrometry

The fractionated CVF samples were prepared for gel electrophoresis by intermixing with SDS sample buffer followed by heating to 95°C in order to denature the proteins. Samples were loaded on a 4–15% Mini-Protean TGX precast gel (Biorad, #456-1086/1083) and SDS gel electrophoresis was performed at 200V. After completion of gel electrophoresis, the proteins in the gel were visualized using a Coomassie stain (Biorad, #1610787) and bands matching the protease-probe complexes were excised and prepared for mass spectrometry. The samples were concentrated with Omix C18MB tips (Agilent Technologies, #A5700310K) and eluted with 5µL buffer B (0.1% formic acid (FA), 65% acetonitrile (ACN)). To each eluted sample, 60µL of buffer A (0.1% FA, water) was added, of which 18 µL were loaded from a 96-well microplate autosampler onto a C18 trap column using the EASY-nLC1000 system (Thermo Fisher Scientific, #LC120) and running buffer C (0.1 % FA in water). The trap column consisted of IntegraFrit capillary (inner diameter 150 µm, New Objective, #IF15010xxxx) cut to 3.2 cm in length and packed in-house with 5µm Pursuit C-18 (Agilent Technologies). Peptides were eluted from the trap column at 300nL/min with an increasing concentration of buffer D (0.1% FA in ACN) over a 30-minute gradient onto a resolving 15 cm long PicoTip Emitter (75µm inner diameter, 8µm tip, New Objective) packed in-house with 3µm Pursuit C-18 (Agilent Technologies). The liquid chromatography setup was coupled online to a Q Exactive Plus (Thermo Fisher Scientific) mass spectrometer using a nanoelectrospray ionization source (Thermo Fisher Scientific) with capillary temperature set to 275°C and spray voltage of 2kV. A 60-minute data-dependant acquisition (DDA) method was setup on the Q Exactive Plus. The full MS1 scan from 400–1500 m/z was acquired in the Orbitrap at a resolution of 70,000 in profile mode with subsequent fragmentation of top 12 parent ions using the HCD cell and detection of fragment ions in the Orbitrap using centroid mode at a resolution of 17,500. The following MS method parameters were used: MS1 Automatic Gain Control (AGC) target was set to 3e6 with maximum injection time (IT) of 100ms, MS2 AGC was set to 1e5 with maximum IT of 50ms, isolation window was 1.6 Da, underfill ratio 2%, intensity threshold 4e4, normalized collision energy was set to 27, charge exclusion was set to fragment only 2+,3+ and 4+ charge state ions, apex trigger was deactivated, peptide match set to preferred and dynamic exclusion set to 45 seconds.

### Data analysis

The mass spectrometry RAW files were uploaded into MaxQuant ver. 1.5.2.8 and searched with built-in Andromeda search engine ^20, 21^. The search was performed with oxidation of methionine and N-terminal protein acetylation as variable modifications, carbamidomethylation of cysteine as a fixed modification, trypsin as the digestive enzyme with a maximum of 2 allowed missed cleavages. The first peptide search tolerance for both samples was 20ppm against a small ‘human first-search’ database for the purpose of mass recalibration, whereas the main search was performed at 4.5 ppm against the Human SwissProt protein database (January 2015 release). The database was reversed to calculate the peptide and protein level false-discovery rate (set at 1%). Proteases were selected from the protein list by comparing the dataset to the Merops database. Candidate active proteases were subsequently selected based on relative protein abundance within the sample, catalytic type, molecular size of the mature protein and substrate specificity.

### Validation of TMPRSS1D in CVF

Validation of TMPRSS11D was performed by preparing 10µg total protein unfractionated CVF sample and SEC fractionated CVF samples for gel electrophoresis as described previously. A total of 20µg HepG2 cell lysate (abcam, #ab7900) was included as a positive control as per manufacturer’s instructions. Following gel electrophoresis and transfer of the proteins to a PDVF membrane, the membrane was blocked in 5% milk in TBS-T at 4°C overnight. The membrane was probed with anti-TMPRSS11D antibody (ab127031, abcam, RRID:AB_11129135) diluted 1 in 500 in 1% milk in TBS-T for 90 minutes at room temperature. Subsequently, the membrane was washed 3 times for 10 minutes with TBS-T and incubated with peroxidase conjugated goat anti rabbit secondary antibody (Jackson Immunoresearch, #111-035-045) diluted 1 in 3000 in 1% milk in TBS-T for 45 minutes. The membrane was washed 4 times for 10 minutes with TBS-T prior to incubation with chemiluminescence substrate, exposure and development.

### Production and purification of mature active KLK13

Recombinant active KLK13 was produced using a *Pichia pastoris* yeast expression system. Briefly, a PCR-amplified DNA fragment encoding the mature KLK13 isoform (36-277 aa) was cloned into the pPIC9 expression vector, in-frame with its α-secretion signal and the alcohol oxidase *AOX1* gene. Purified mature *KLK13* pPIC9 DNA construct was confirmed by sequencing using the 5’- *AOX1*, 3’ *AOX1* and the α-secretion signal vector-specific primers and the NCBI BLAST align program. The KLK13-pPIC9 construct was linearized with SacI enzyme and transformed into the KM71 *P. pastoris* strain via electroporation. A stable KM71 transformant was grown in 1 liter of buffered glycerol-complex medium (BMGY) media. After 2 days, yeast culture was centrifuged, and the cell pellet was resuspended in 300 ml of BMMY media ( *A*600 = 10). Recombinant KLK13 expression was induced with 1% methanol for 5 days at 30°C in a shaking incubator (250 rpm). Recombinant KLK13 was purified from culture supernatant by ultraconcentration, serial dialysis, and centrifugation procedures, followed by cation-exchange chromatography using an automated ÄKTA FPLC system on a pre-equilibrated 5-ml cation-exchange HiTrap high performance Sepharose HP-SP column (GE Healthcare). Recombinant KLK13 was further purified using 10mL cation-exchange Source15S TricornTM column (GE Healthcare). The collected fractions were pooled, concentrated, analyzed by SDS-PAGE and Western blotting and stored at −80°C until further use. The concentration of purified protein was measured using a Coomassie protein assay and KLK13 ELISA as described previously ^22^. The N-termini of the bands identified on SDS-PAGE gels were analyzed with N-terminal Edman sequencing.

### Validation of KLK13 in CVF

Unfractionated CVF and SEC fractionated CVF samples were prepared for gel electrophoresis as described previously. A total of 1µg in-house produced recombinant KLK13 was included as a positive control. Following gel electrophoresis and protein transfer, the membrane was blocked in 5% milk in TBS-T at 4°C overnight. The membrane was probed with a in-house produced monoclonal anti-KLK13 antibody diluted 1 in 50 in 1% milk in TBS-T for 90 minutes at room temperature ^23^. Subsequently, the membrane was washed 3 times for 10 minutes with TBS-T and incubated with peroxidase conjugated goat anti mouse secondary antibody (Jackson Immunoresearch, #115-035-146) 1 in 3000 diluted in 1% milk in TBS-T for 45 minutes. The membrane was washed 4 times for 10 minutes with TBS-T prior to incubation with chemiluminescence substrate (GE healthcare, #45000875), exposure and development.

An immunocapture method was developed to confirm the presence and activity of KLK13 in CVF. In this assay, 500ng of in-house produced monoclonal KLK13 antibody was coated overnight on a 96-well plate in coating buffer (50mM Tris, 0.05% Tween-20, pH 7.8). The plate was washed 3 times with washing buffer (50mM Tris, 150mM NaCl, 0.05% Tween-20, pH 7.8) and blocked with 1% bovine serum albumin (BSA) in activity buffer (100mM NaH _2_PO _4_/Na _2_HPO _4_, pH 8.5) for 90 minutes. The plate was washed 3 times with washing buffer prior to incubation with CVF samples (N=3) in a 1 in 5, 1 in 10 and 1 in 50 dilutions for 3 hours at room temperature. Activity buffer without active enzyme, IgG coated wells, and 6nM in-house produced recombinant KLK13 were included as a negative and positive controls respectively. The wells were washed extensively 6 times with washing buffer to remove any unbound protein. A final 0.5mM VPR-AMC substrate (Bachem, #4003460) was added to the wells and the resulting increase in fluorescence was measured in 1-minute intervals on a Wallac Envision fluorometer set at 355nm for excitation and 460nm for emission. Proteolytic KLK13 activity in CVF was semi-quantified by comparing the rate of substrate hydrolysis after subtraction of the fluorescence of enzyme-free reactions to the recombinant KLK13 activity.

The influence of pH on the proteolytic activity of KLK13 was tested by measuring the cleavage of fluorogenic substrate by 6nM recombinant KLK13 in buffer with pH ranging from 3.5 to 10.0 in 0.5 increments. Fluorescence was measured every minute for 15 minutes on a Wallac Envision fluorometer (Perkin-Elmer) set at 355nm for excitation and 460nm for emission. Proteolytic activity was quantified by calculating the rate of substrate hydrolysis after subtraction of the fluorescence of enzyme-free reactions relative to proteolytic activity at pH 8.0. All reactions were performed at 37°C in triplicate on 2 separate days.

## Results

### Investigation of proteolytic activity in CVF

**Figure 1.**
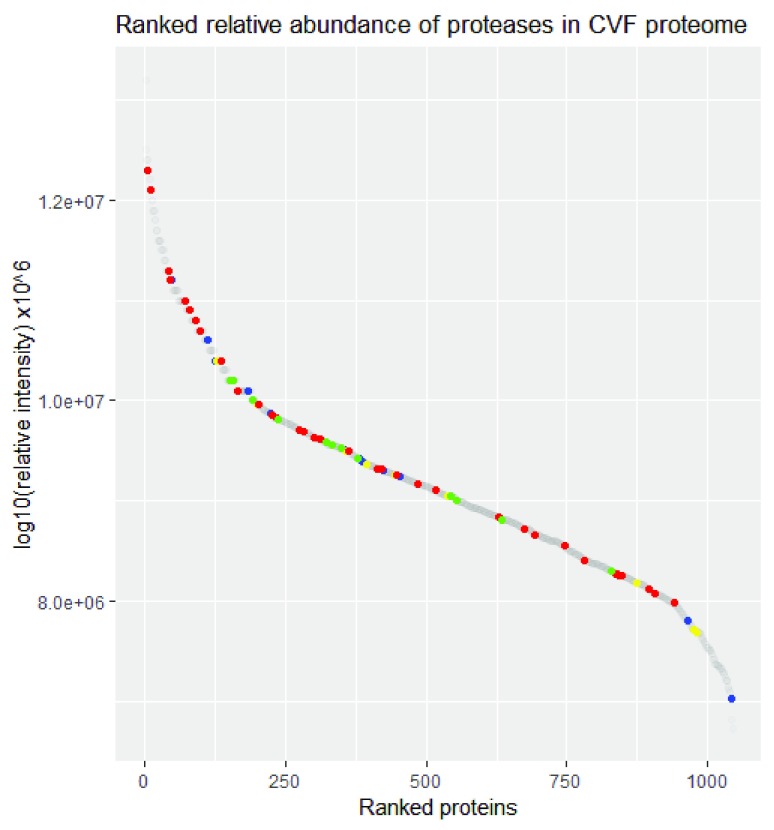
The ranked relative abundance of proteases in the cervical-vaginal fluid (CVF) proteome. Visualization of the ranked relative abundance of all proteins in CVF (1,065 proteins, grey dots) highlighting serine proteases (36 proteins, red dots), cysteine proteases (13 proteins, blue dots), threonine proteases (13 proteins, green dots) metalloproteinases (10 proteins, yellow dots). Aspartic acid proteases (1 protein), mixed catalytic type proteases (3 proteins) and unassigned proteases (4 proteins) were also identified in CVF.

**Figure 2.**
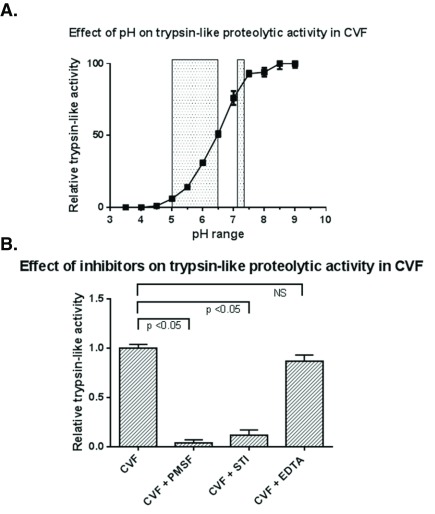
Trypsin-like proteolytic activity in cervical-vaginal fluid (CVF). ( **A**) Trypsin-like proteolytic activity in CVF is highly pH dependent as demonstrated by the cleavage of fluorogenic trypsin-like VPR-AMC substrate by proteases in CVF. Grey areas represent the CVF pH during infections and post-menopausal status (pH 5–6.5) and after sperm ejaculation (pH 7.2) Trypsin-like activity is shown as relative to maximum activity at pH 9.0 ( **B**) Trypsin-like proteolytic activity is significantly inhibited by PMSF and STI, whereas EDTA has no significant effect. The trypsin-like activity is shown as relative to the activity observed in uninhibited CVF.

A total of 85 proteases were identified in the CVF proteome based on samples from healthy, non-pregnant women of reproductive age ^7^. As shown in Figure 1, proteases of different catalytic classes are present in the entire relative abundance spectrum of the CVF proteome. Multiple serine proteases rank among the most abundant proteins in CVF. As can be seen in Figure 2A, the trypsin-like proteolytic activity in CVF is optimal at slightly alkaline pH levels (pH 7.5-9). Almost half of the maximal trypsin-like proteolytic activity is CVF remains at pH 6.0, whereas there is negligible trypsin-like proteolytic activity at pH 4.5 or below. The presence of PMSF and STI significantly reduced the trypsin-like proteolytic activity in CVF (p<0.05), whereas EDTA did not impact the proteolytic activity in CVF significantly ( Figure 2B).

### Visualization of active, trypsin-like proteases in CVF

**Figure 3.**
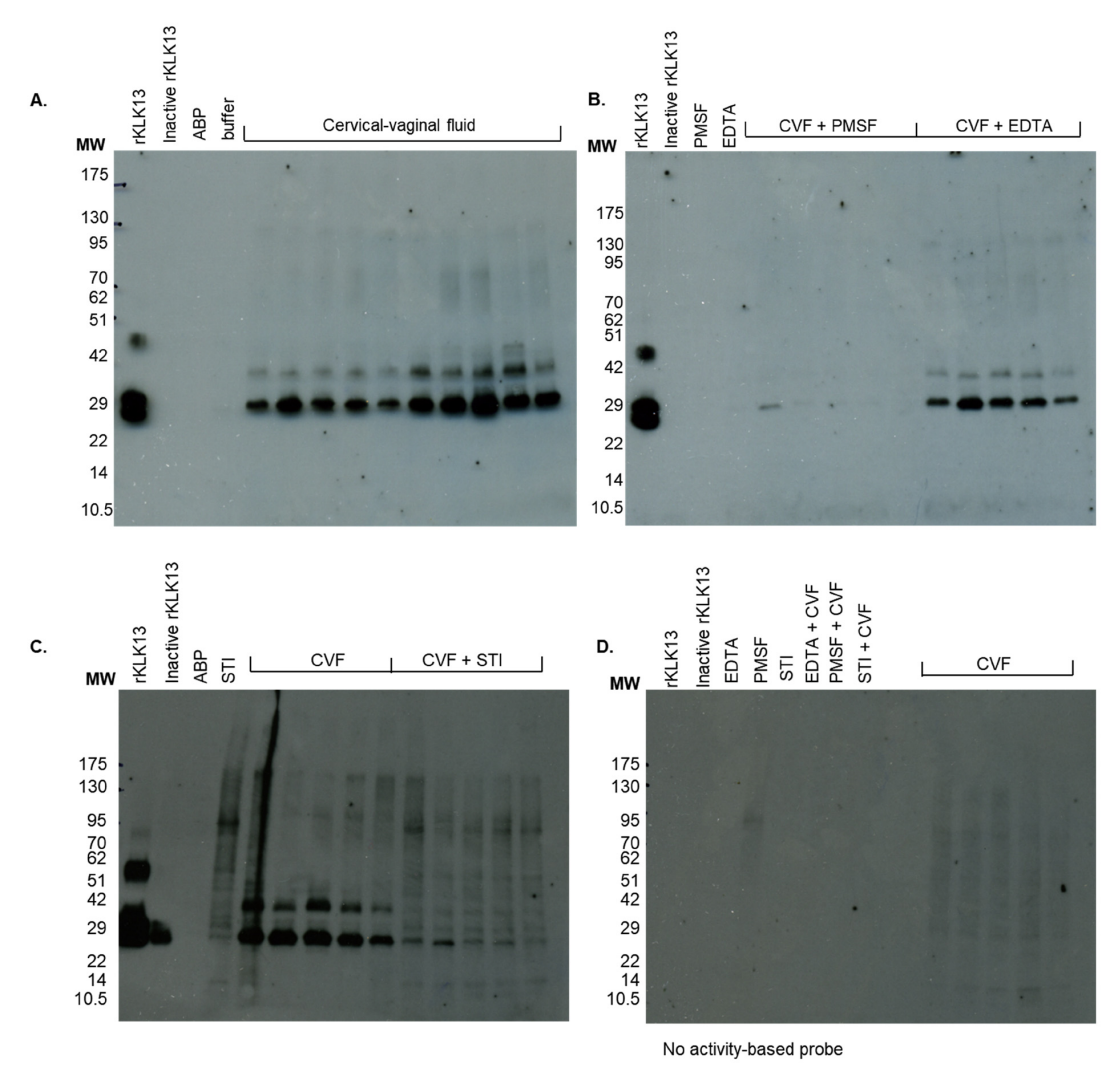
Visualization of active trypsin-like proteases in cervical-vaginal fluid (CVF). ( **A**) Active trypsin-like proteases in CVF (N=10) were coupled to the biotinylated ABP probe and visualized following gel electrophoresis. Trypsin-like proteolytic activity was present in two separate bands (28kDa and 35kDa) in each of the samples. ( **B**) Co-incubation of CVF with PMSF prior to coupling to the ABP probe resulted in the inhibition of active trypsin-like proteolytic activity, whereas co-incubation with EDTA did not effect active trypsin-like proteolytic activity ( **C**) Incubation with STI prior to labeling CVF with the ABP probe inhibited the trypsin-like proteolytic activity in CVF compared to the control condition ( **D**) Negative control condition incubated without ABP probe showed no trypsin-like proteolytic activity in experimental samples.

Active proteases present in CVF from healthy, non-pregnant women of reproductive age were visualized using the ABP probe, which binds trypsin-like active proteases in a covalent and specific manner ^19^. As illustrated in Figure 3A, trypsin-like protease activity in CVF is present at 2 separate molecular weight (MW) bands at approximately 28 and 35kDa. Apart from differences in the intensity of the bands, the observed expression pattern was nearly identical among the different CVF samples. Next, we investigated whether the trypsin-like protease activity in CVF can be attributed to proteases of certain catalytic types. Complete cessation of trypsin-like activity in CVF was accomplished by incubation of the CVF samples with the irreversible protease inhibitor PMSF, targeting both serine and cysteine proteases, prior to coupling to the ABP ( Figure 3B). Additionally, incubation of CVF with reversible serine protease inhibitor STI resulted in a near complete inhibition of the protease activity in CVF compared to the uninhibited control condition ( Figure 3C). Inhibition of metalloproteinases via incubation with EDTA did not affect the trypsin-like proteolytic activity in CVF ( Figure 3B). As expected, no active proteases were detected in the control condition without probe addition ( Figure 3D).

### Identification of active proteases TMPRSS11D and KLK13

**Figure 4.**
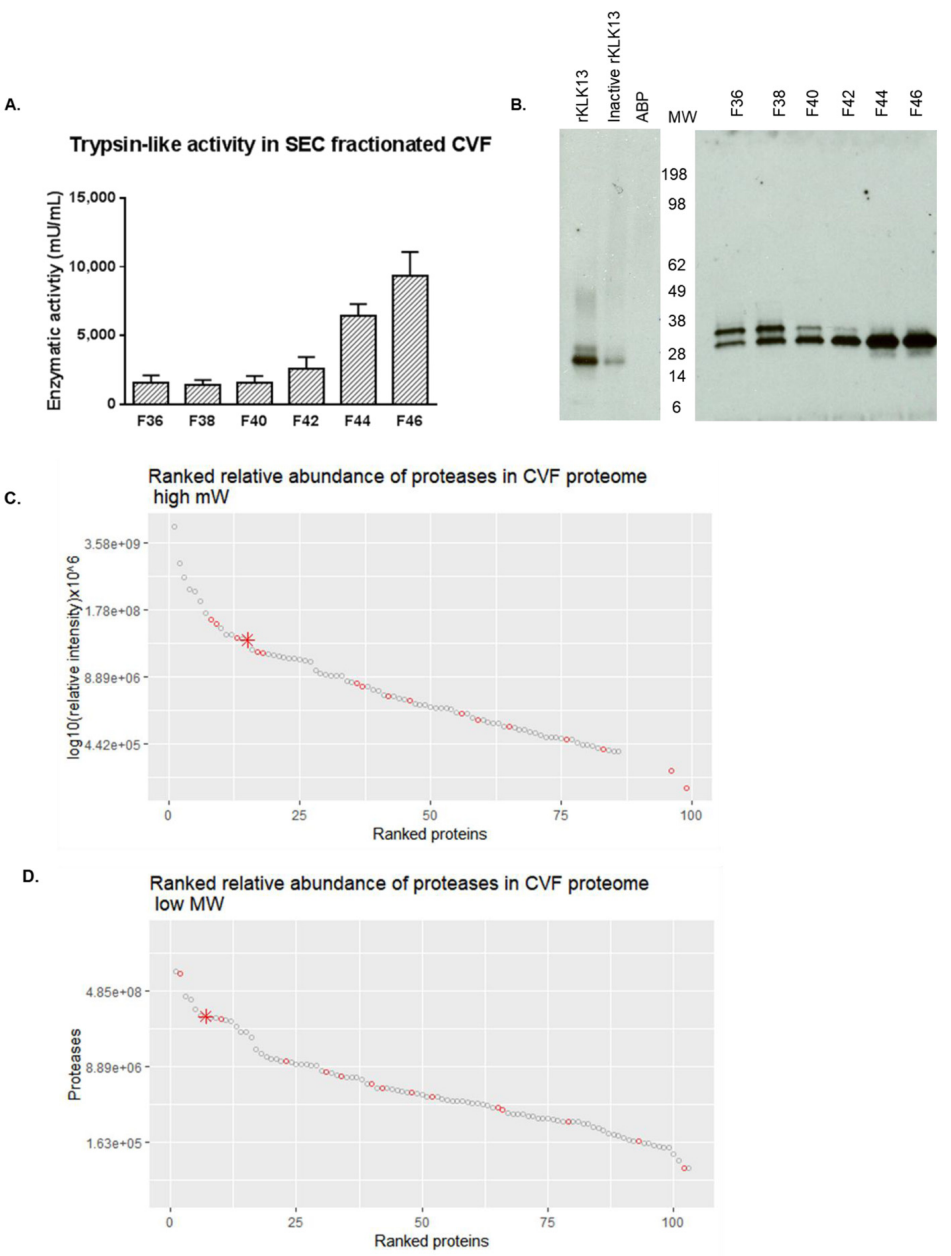
Trypsin-like proteolytic activity in size exclusion (SEC) fractionated cervical-vaginal fluid (CVF). ( **A**) trypsin-like activity was quantified in fractions F36 – F46 in SEC fractionated CVF using VPR-AMC substrate ( **B**) Visualization of separation of high (F36-F38) and low (F42-F46) MW band containing trypsin-like proteolytic activity in SEC fractionated CVF ( **C**) Ranked relative abundance based on average MS1 area of proteases (red dots, KLK13 as star) in high MW fractions across CVF proteome ( **D**) Ranked relative abundance based on average MS1 area of proteases (red dots, TMPRSS11D as star) in low MW fractions across CVF proteome. For more comments see text.

SEC fractionation of CVF resulted in the successful separation of the high and low MW proteases as demonstrated by the split in proteolytic activity measured in fractionated CVF using the fluorogenic VPR-AMC substrate ( Figure 4A) and subsequent visualization of active proteases in CVF using the ABP ( Figure 4B). Both the high and low MW bands were excised and resolved separately with mass spectrometry resulting in the identification of approximately 100 proteins for the high MW and low MW bands ( Figure 4C–D). Candidate active proteases were selected based on relative protein abundance among the sample proteome, serine proteolytic catalytic type, correct molecular size and trypsin-like substrate specificity. This approached resulted in the identification of KLK13 as a candidate protease for the high MW band and TMPRSS11D as the candidate active protease in the low MW band.

### Validation of TMPRSS11D and KLK13 in CVF

**Figure 5.**
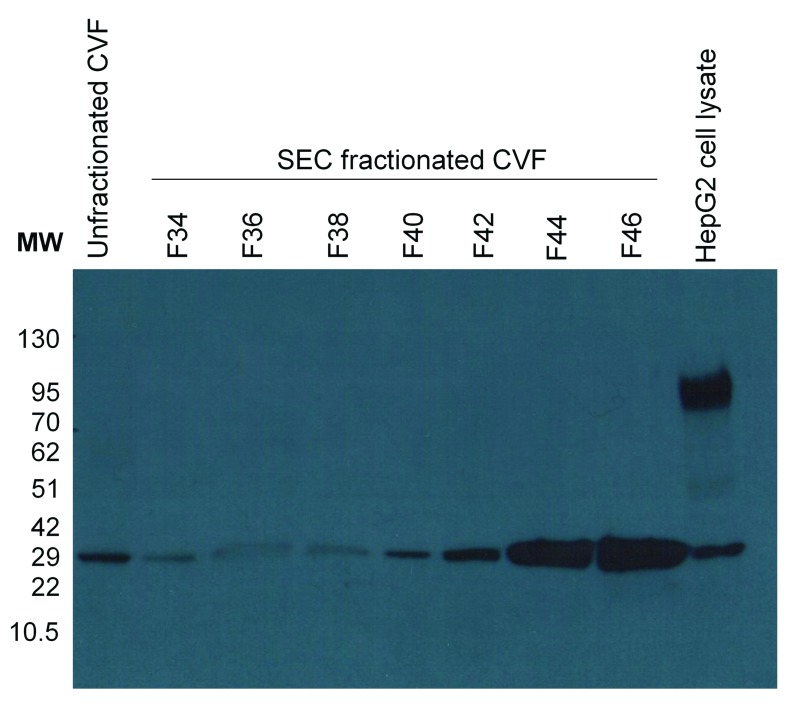
Validation of TMPRSS1D in cervical-vaginal fluid (CVF). TMPRSS11D is detected in unfractionated CVF and in F42-F46 of SEC fractionated CVF corresponding with the proteolytic activity visualized with the ABP in the low MW band. HepG2 cell lysate is included as a positive control.

A Western blot showed TMPRSS11D at the correct MW in unfractionated CVF samples ( Figure 5). Furthermore, TMPRSS11D is detected in the CVF fractions corresponding to the low MW band as expected.

**Figure 6.**
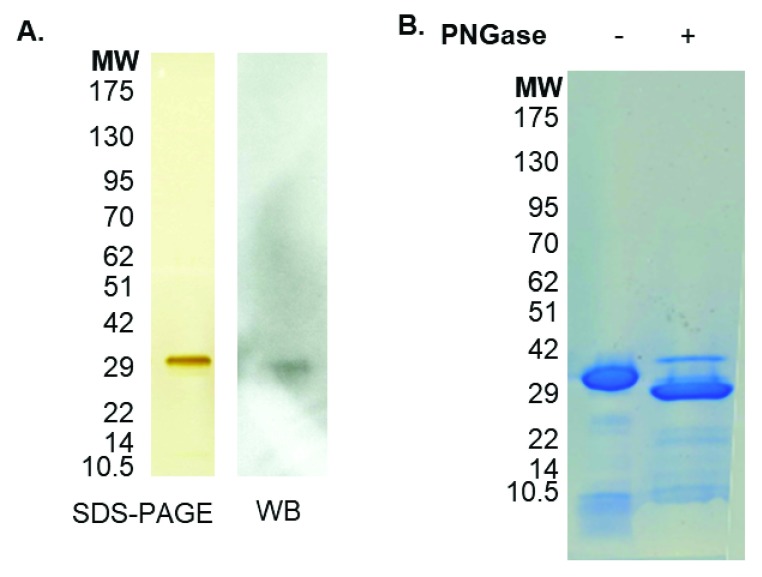
Production of recombinant active KLK13 protein. ( **A**) Silver staining of reduced SDS-PAGE of KLK13 following purification with band visible at 30kDa. The band was found to contain KLK13 sequences by mass spectrometry and recognized by anti-KLK13 antibody by western blot (WB) ( **B**) Coomassie staining of reduced SDS-PAGE of purified KLK13 showing PNGase F treatment increasing the 28kDa band intensity.

Mature, active recombinant KLK13 was produced in a *P. pastoris* expression system. The KLK13 750bp PCR construct was successfully ligated into a eukaryotic pPIC9 vector as confirmed by DNA sequencing. The pPIC9- *klk13* vector was subsequently linearized by SacI enzyme digestion and electroporated into *P. pastoris* KM71 cells. Gel electrophoresis of the KLK13 protein, isolated from the methanol induced yeast cell culture medium in a multi-step purification and concentration process, resulted in the visualization of KLK13 ( Figure 6A). PNGaseF treatment revealed that the higher MW band is the result of glycosylation at 225N. ( Figure 6B). KLK13 identity was confirmed by mass spectrometry analysis.

**Figure 7.**
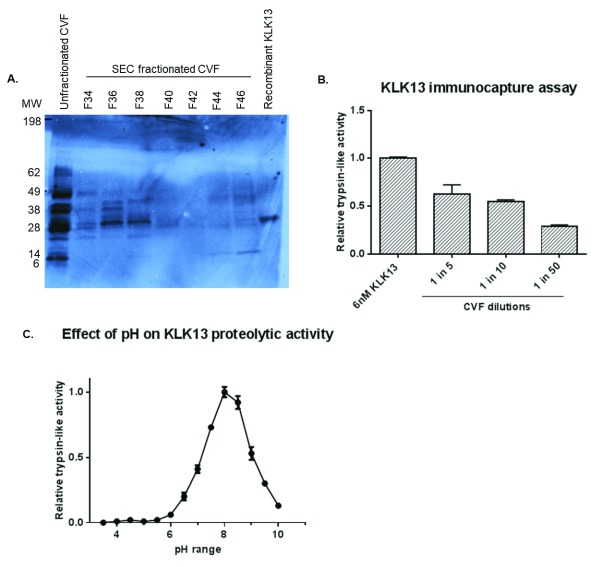
Validation of KLK13 presence in cervical-vaginal fluid (CVF). ( **A**) Western blot with KLK13 monoclonal antibody detected bands with multiple molecular sizes in unfractionated CVF. KLK13 was detected as a single band around 30-35kDa in SEC fractionated CVF F36-38 corresponding with the proteolytic activity visualized with the ABP probe in the high MW band. In-house produced recombinant KLK13 was included as a positive control ( **B**) Immunocapture assay demonstrating the dilution dependent presence of KLK13 in CVF in active form and not bound to endogenous inhibitors ( **C**) Effect of pH on KLK13 trypsin-like proteolytic activity showing pH dependency with optimum activity observed at pH 8.0.

KLK13 is present in unfractionated CVF at multiple sizes that correspond with a smaller, potentially degraded form of KLK13, KLK13 bound to endogenous inhibitors and KLK13 in its active mature form ( Figure 7A). Analysis of the fractionated CVF sample demonstrated that the presence of KLK13 at the correct MW in the fractions corresponding with the high MW band of Figure 3 and Figure 4. The cleavage of the fluorescent substrate by immunocaptured KLK13 showed that at least a proportion of KLK13 is present in CVF in its mature, active form, which is not bound to endogenous inhibitors ( Figure 7B). The proteolytic activity of recombinant KLK13 was demonstrated to be highly pH-dependent with maximum activity observed at pH 8.0 and minimal activity at the physiological pH observed in CVF (<6) ( Figure 7C).

## Discussion

In-depth proteomic analysis of CVF of healthy, non-pregnant women previously revealed the presence of numerous proteases, including a large number of serine proteases, in this fluid. Indeed, multiple serine proteases are among the most abundant proteins in the CVF proteome and incubation of CVF with serine protease inhibitors significantly decreased proteolytic activity in CVF. Both findings highlight the functional importance of the serine protease family in CVF.

In the current study, we performed a functional proteomic approach coupling a biotinylated ABP probe with trypsin-like specificity with mass spectrometry, to profile the active proteases in CVF. This powerful approach resulted in the identification of serine proteases KLK13 and TMPRSS11D as putative active proteolytic enzymes in CVF.

TMPRSS11D, better known as human airway trypsin-like protease or HAT, displays trypsin-like activity and belongs to the type II transmembrane serine proteases ^24^. TMPRSS11D is synthesized as an inactive zymogen which requires proteolytic cleavage to release the soluble extracellular 27kDa mature active form ^25, 26^. This transmembrane protein is expressed in the squamous epithelial cells of the vagina and the glandular cells of the endometrium and was detected in the CVF proteome ^7, 27^. Mostly studied in the context of respiratory diseases, TMPRSS11D is a versatile protein whose functions include fibrinolysis, PAR-2 receptor activation and the processing of viral glycoproteins ^28^. Interestingly, activation of the PAR-2 receptor negatively affects the structural integrity of the vaginal epithelium prompting the hypothesis that TMPRSS11D is involved in barrier function modulation in the lower female reproductive system ^29– 32^. Additionally, TMPRSS11D is differentially expressed in the peri-implantation uterine luminal epithelium in mice where it is putatively involved in morphological changes required for the initial attachment of the embryo to the luminal epithelium ^33^. It is unknown whether TMPRSS11D plays a similar role in the human endometrial environment.

KLK13 belongs to the kallikrein-related peptidase family; a group of 15 secreted serine proteases with either tryptic or chymotryptic substrate specificity. KLKs are expressed throughout the human body with the highest expression levels observed in the fluids and tissues of the male and female reproductive systems ^34, 35^. Studies on KLK13 have predominantly focused on its applicability as a cancer biomarker, leaving the physiological role of this protein not well understood ^36, 36– 40^. *In vitro* assays showed that despite being present in active form and unbound to endogenous inhibitors, KLK13 activity will largely be inhibited due to the low pH in CVF.

KLK13 is the highest abundant KLK member among a cassette of KLKs that are co-expressed in CVF ^34^. The concentration of KLK13 in CVF is approximately 12 mg/L (data not shown); only KLK2 and KLK3 are found at higher concentrations in the male reproductive system ^41^. Both KLK5 and KLK12 have been studied in the lower female reproductive tract and are thought to be involved in the desquamation of the vaginal epithelium, facilitation of sperm transport towards the oocyte and the processing of antimicrobial peptides in CVF ^42, 43^. KLK13 could also be involved in these processes either directly or, based on the detection of multiple members of the KLK family in CVF, via participation in a proteolytic cascade in which proteases activate consecutive proteases thereby amplifying the proteolytic potential ^44^.

In conclusion, the current study provides insights on the proteolytic functioning in the lower female reproductive tract. Fitting in the framework that serine proteases are the predominant source of trypsin-like proteolytic activity in CVF, ABP proteomics resulted in the identification of KLK13 and TMPRSS11D as candidate active proteases in this complex biological fluid. Both proteolytic enzymes exert their effects extracellularly in CVF which led us to believe that the activity of these enzymes will be largely inhibited by the low pH ^45^. Based on the highly pH-dependent trypsin-like proteolytic activity in this complex biological fluid, the possibility that pH acts as a regulatory mechanism to control proteolytic activity in CVF was raised. As can be seen by the grey overlay in Figure 2A, proteolytic activity is absent at physiological pH levels (4.5 or below). It is possible however that conditions characterized by an elevated pH would allow for moderate to high levels of protease activity, and more specifically KLK13 activity, in CVF. It is known that the vaginal pH shows a dramatic rise to 7.2 within seconds after sperm ejaculation ^46^. It is therefore not unlikely that KLK13 could be activated just after ejaculation during sexual intercourse, participates in a proteolytic cascade with proteases present in the ejaculate and initiates events associated with fertility. Additionally, CVF pH is elevated during certain vaginal infections and in postmenopausal women ^47, 48^. These conditions provide a window of opportunity for proteolytic activity in the lower female reproductive system. The current study provides a framework for future investigations aimed at understanding proteolytic functioning within the complexities of the lower female reproductive system.

## Data availability

Underlying data is available from figshare:

Dataset 1. Ranked relative abundance proteases in CVF. https://dx.doi.org/10.6084/m9.figshare.7137920.v1 ^49^

Dataset 2. Trypsin-like proteolytic activity in CVF. https://dx.doi.org/10.6084/m9.figshare.7137938.v1 ^50^

Dataset 3. Visualization of active trypsin-like proteases in CVF. https://dx.doi.org/10.6084/m9.figshare.7137941.v1 ^51^

Dataset 4. Trypsin-like proteolytic activity in size exclusion (SEC) fractionated CVF. https://dx.doi.org/10.6084/m9.figshare.7137944.v1 ^52^

Dataset 5. Validation of TMPRSS1D in CVF. https://dx.doi.org/10.6084/m9.figshare.7137947.v1 ^53^

Dataset 6. Production of recombinant active of protein KLK13 protein. https://dx.doi.org/10.6084/m9.figshare.7137953.v1 ^54^

Dataset 7. Validation of KLK13 presence in CVF. https://dx.doi.org/10.6084/m9.figshare.7137962.v1 ^55^

All data is available under a CC by 4.0 Universal licence
