# Supplementary material for: Functional proteomic profiling reveals KLK13 and TMPRSS11D as active proteases in the lower female reproductive tract
Source: F1000Res. 2018 Dec 31;7:1666. Originally published 2018 Oct 19. [Version 2] doi: 10.12688/f1000research.16255.2 (PMC6329257; doi:10.12688/f1000research.16255.2)
Supplement: Peer review history

# Peer review: Referee response for version 2

**DOI:** 10.5256/f1000research.19125.r42238

**Published:** 2019-01-09

**Authors:** Céline Deraison

**Affiliations:** 1 Digestive Health Research Institute (IRSD), U1220, INSERM, Toulouse, France

Thanks for clarification and changes.

I have read this submission. I believe that I have an appropriate level of expertise to confirm that it is of an acceptable scientific standard.

# Peer review: Referee response for version 1

**DOI:** 10.5256/f1000research.17754.r40431

**Published:** 2018-11-22

**Authors:** Céline Deraison

**Affiliations:** 1 Digestive Health Research Institute (IRSD), U1220, INSERM, Toulouse, France

The presented study aimed to characterize active serine proteases in cervicovaginal fluid (CVF). Active serine proteases were profiled using activity based probe from human CVF highlighting 2 close bands. A whole proteomic strategy was performed using human CVF and 2 proteases were deeper characterised, KLK3 and TMPRSS11D.

 CVF is a particular fluid since the pH is very low and also numerous proteases. The study is well designed and based on human samples, increasing the interest of the study. The manuscript is well written. However different points will need to be discussed or explained.

  

1. How could we explain why using ABP only two bands are highlighted (figure3) since 36 serine proteases were identified on MS/MS study (Figure1)?
2. A clear correlation between activity of KLK13 and TMPRSS11D and their presence in CVF is not well established. Why authors did not perform MS/MS approach on active proteases enriched sample after ABP trapping? 
3. Authors claimed that KLK3 active form was present in CVF sample. However in figure 3 and 4 it not clear that immunoband from recombinant active KLK13 and higher band from ABP treated CVF are at the same size. In addition, why active recombinant KLK3 showed two bands after ABP trapping? Why inactive mutant KLK13 is also trapped by ABP? What is the identity of mutation? Why KLK13 is always present on gels in figure 3? Authors suggested that KLK13 is active in CVF sample since proteolytic activity is detected with immunocaptured fraction using antibody against KLK13. “The cleavage of the fluorescent substrate by immunocaptured KLK13 showed that at least a proportion of KLK13 is present in CVF in its mature, active form, which is not bound to endogenous inhibitors.In addition, the used antibody was produced in the lab. Could authors provide a bibliographic reference illustrating the specificity of the antibody since the group of KLK contains some very similar proteins.
4. In figure 4, it could be informative to highlight KLK13 and TMPRSS11D on graphs. On this figure, it is not clear how is ranked the intensity of each protein. Does it correspond to the quantity of peptides identified by MS/MS for each protein?
5. In discussion section, 2 sentences implied that a direct identification of active protease targeted by ABP was performed. Please, correct these sentences (‘This powerful approach resulted in the identification of serine proteases KLK13 and TMPRSS11D as major active proteolytic enzymes in CVF. ABP proteomics resulted in the identification of KLK13 and TMPRSS11D as active proteases in this complex biological fluid.)

 Minor comments:

- Figure 2: The graph represents the “relative trypsin-like activity”. What is the reference?
- What does NRT mean? 
- The biological concentration of KLK13 is compared to KLK2 and KLK3 in sperm (“The concentration of KLK13 in CVF is approximately 12 mg/L (data not shown); only KLK2 and KLK3 are found at higher concentrations in the male reproductive system). It would be interesting to support this sentence with a bibliographic reference.

I have read this submission. I believe that I have an appropriate level of expertise to confirm that it is of an acceptable scientific standard, however I have significant reservations, as outlined above.

# Response

**Published:** 2018-12-08

**Authors:** Eleftherios P Diamandis

**Affiliations:** Mount Sinai Hospital, Toronto, Canada

1. **How could we explain why using ABP only two bands are highlighted (figure3) since 36 serine proteases were identified on MS/MS study (Figure1)?**

The ABP recognizes only active serine proteases, whereas the MS/MS study aims to identify all proteases in CVF regardless of their functional status. The difference in the number of identified proteases (36 in MS/MS versus 2 clusters using the ABP) is most likely due to state of the proteases. A number of proteases will be present in CVF, but in zymogen form or closely bound to endogenous inhibitors. These proteases will be identified using MS, but would not be visible on the ABP blot since these proteins do not exert proteolytic activity.

1. **A clear correlation between activity of KLK13 and TMPRSS11D and their presence in CVF is not well established. Why authors did not perform MS/MS approach on active proteases enriched sample after ABP trapping? **

The availability of the ABP was a limiting factor in this study thereby preventing us to perform MS/MS on an ABP-enriched samples. We aimed to establish the correlation by showing that TMPRSS11D is present in CVF at the correct MW and fractionated CVF in the expected fractions. For KLK13, we also showed that KLK13 is present in CVF and fractionated CVF at the correct MW and in the expected fractions. Additionally, we using the immunocapture assay we demonstrated that (a proportion of) KLK13 is present in CVF in active form without its prodomain or bound to endogenous inhibitors.

  

1. **Authors claimed that KLK3 active form was present in CVF sample. However in figure 3 and 4 it not clear that immunoband from recombinant active KLK13 and higher band from ABP treated CVF are at the same size. In addition, why active recombinant KLK3 showed two bands after ABP trapping? Why inactive mutant KLK13 is also trapped by ABP? What is the identity of mutation? Why KLK13 is always present on gels in figure 3? Authors suggested that KLK13 is active in CVF sample since proteolytic activity is detected with immunocaptured fraction using antibody against KLK13. “The cleavage of the fluorescent substrate by immunocaptured KLK13 showed that at least a proportion of KLK13 is present in CVF in its mature, active form, which is not bound to endogenous inhibitors.In addition, the used antibody was produced in the lab. Could authors provide a bibliographic reference illustrating the specificity of the antibody since the group of KLK contains some very similar proteins.**

The data in figure 3 and 4 demonstrating KLK13 in CVF is supported by additional evidence from the mass spectrometry analysis indicating that KLK13 is abundantly present in the bands corresponding to the higher MW band from the ABP-treated CVF. The active recombinant KLK13 shows 2 bands after ABP trapping most likely due to glycosylation. This affects the MW of the protein, while at the same time still allowing the activity of the protease. KLK13 is visible as 2 bands: one glycosylated and one not glycosylated.

 A logical explanation for the presence of KLK13 throughout the gel in figure 4 is due to the high abundance of this protein in CVF. It is frequently observed that high abundance proteins will not separate clearly in the expected fractions, but rather are present throughout the fractions. We expect that this is the case for KLK13 in fractionated CVF.

 KLK13 was inactivated not by introducing a mutation, but via incubation with the potent irreversible serine protease inhibitor PMSF. There are two explanations that clarify the small band present in the inactivated KLK13. It is possible that the band in inactive KLK13 is due to the proximity to the active recombinant KLK13 and is a result of spillover from the KLK13 lane into the inactive KLK13 lane. Alternatively, it is possible that KLK13 was not completely inactivated by PMSF resulting in the greatly diminished band shown on the blot.

 We have provided the reference in the manuscript for the production of the monoclonal KLK13 antibody used in this study.

  

1. **In figure 4, it could be informative to highlight KLK13 and TMPRSS11D on graphs. On this figure, it is not clear how is ranked the intensity of each protein. Does it correspond to the quantity of peptides identified by MS/MS for each protein?**

 

 We agree that figure 4 will be improved by highlighting KLK13 and TMPRSS11D. We have adjusted the figure in the manuscript accordingly. The proteins were ranked according to the intensity of each protein for which we used the average MS1 area of each protein as a proxy measure of protein abundance. We have adjusted the legend of the figure to reflect this change.

  

1. **In discussion section, 2 sentences implied that a direct identification of active protease targeted by ABP was performed. Please, correct these sentences (‘This powerful approach resulted in the identification of serine proteases KLK13 and TMPRSS11D as major active proteolytic enzymes in CVF. ABP proteomics resulted in the identification of KLK13 and TMPRSS11D as active proteases in this complex biological fluid.)**

We agree with the reviewer and have changed these sentences in the manuscript accordingly.

  Minor comments:

- **Figure 2: The graph represents the “relative trypsin-like activity”. What is the reference?**

For figure 2A the trypsin-like activity is relative to the trypsin-like activity measured at pH 9.0. This is the pH at which maximum trypsin-like activity was observed. In figure 2B, the trypsin-like activity is presented as relative to the activity in CVF without any inhibitors. We have adjusted the wording in the legend to better reflect this.

  

- **What does NRT mean? **

The 225NRT stands for the N-R-T amino acids in the KLK13 sequence. We have changed the wording in the manuscript to reflect that the glysocylation site is 225N.

  

- **The biological concentration of KLK13 is compared to KLK2 and KLK3 in sperm (“The concentration of KLK13 in CVF is approximately 12 mg/L (data not shown); only KLK2 and KLK3 are found at higher concentrations in the male reproductive system). It would be interesting to support this sentence with a bibliographic reference.**

 

 We have included the appropriate reference to support the biological concentrations of KLKs in the CVF and seminal plasma.

# Peer review: Referee response for version 1

**DOI:** 10.5256/f1000research.17754.r39959

**Published:** 2018-11-01

**Authors:** Georgios Pampalakis

**Affiliations:** 1 Department of Pharmacy, School of Health Sciences, University of Patras, Patras, Greece

The study uses functional proteomics and specifically Activity-Based Probe proteomics to identify the active proteases in cervicovaginal fluid (CVF). CVF is an interesting biological fluids because it contains high level of protease (and up to now their activation status was not known) and very low pH (<4.5).

 The study is well designed. There are a few minor comments that will help the readers to understand better the findings.

 Specifically: 

1. What is the structure of the ABP used in the present study? (the ref 19 describes the synthesis of more than 1 ABP for probing trypsin-like activities).
2. For their proteomic identification of ABP-targets, the authors cut the gel at the position of ABP signaling and performed a mass spectrometry analysis. In their analysis why did the authors not try to identify the tryptic fragment that contains the active-site serine covalently attached to the ABP? In this way, the authors could identify other potential proteases that bind the ABP (therefore they are active in the sample).
3. In Figure 3C why does the inactive KLK13 sample show a signal?
4. What is the nature of inactive KLK13 (e.g. mutant at active site Ser?)

I have read this submission. I believe that I have an appropriate level of expertise to confirm that it is of an acceptable scientific standard.
